# Prevalence of the single-nucleotide polymorphism rs11554137 (IDH1^105GGT^) in brain tumors of a cohort of Italian patients

**DOI:** 10.1038/s41598-018-22222-y

**Published:** 2018-03-13

**Authors:** Giorgia Acquaviva, Michela Visani, Dario de Biase, Gianluca Marucci, Enrico Franceschi, Alicia Tosoni, Alba A. Brandes, Kerry J. Rhoden, Annalisa Pession, Giovanni Tallini

**Affiliations:** 1 Department of Medicine (Dipartimento di Medicina Specialistica, Diagnostica e Sperimentale) - Molecular Diagnostic Unit, Azienda USL di Bologna, University of Bologna School of Medicine, Bologna, Italy; 2 Department of Pharmacy and Biotechnology (Dipartimento di Farmacia e Biotecnologie) - Molecular Diagnostic Unit, Azienda USL di Bologna, University of Bologna, Bologna, Italy; 3 Anatomic Pathology Unit, AUSL of Bologna, Bologna, Italy (currently at Department of Neuropathology, IRCCS Foundation Carlo Besta Neurological Institute, Milan, Italy; 4 Department of Oncology, AUSL Bologna – IRCCS Institute of Neurological Sciences, Bologna, Italy; 5 Medical Genetics Unit, Department of Medical and Surgical Sciences (DIMEC), University of Bologna, Bologna, Italy

## Abstract

*IDH* mutational status is required for proper diagnosis according to the WHO criteria revised in 2016. The single nucleotide polymorphism (SNP) rs11554137 (*IDH1*^105GGT^) at codon 105 of *IDH1* has been reported in patients with several tumor types, including those with glioma. The aim of this study is to investigate the prevalence of *IDH1*^105GGT^ in a cohort of brain tumors, and its association with clinicopathologic features and *IDH1* and *IDH2* missense mutations. Exon 4 of *IDH1* and *IDH2* was analyzed in a series of brain tumors classified according to current WHO criteria. DNA from control individuals was analyzed to infer the prevalence of *IDH1*^105GGT^ in the reference population. Analysis was performed using next generation sequencing. *IDH1*^105GGT^ was three times more frequent in patients with tumors (44/293 cases, 15.0%) vs. population controls (6/109, 5.5%) (*p* = *0*.*0102*). *IDH1*^105GGT^ was more frequent in grade III tumors (26.1%) compared to grade II (10.9%, p = 0.038) and grade IV tumors (13.7%, p = 0.041). *IDH1*^*105GGT*^ was more frequent in grade II and III tumors without an *IDH* tumor missense mutation (43.8%) than in those with (11.5%, p = 0.005). The *IDH1*^105GGT^ SNP likely represents an important genetic marker, worthy of additional investigation to better understand the clinical and biological features of *IDH*-WT infiltrating gliomas.

## Introduction

The isocitrate dehydrogenase (IDH) family includes three isozymes (IDH1, IDH2, IDH3) involved in the Krebs cycle as active participants in NADPH production. These proteins also play an important role in the cellular control of oxidative damage^1,2^. The IDH1 protein is localized to the cytoplasm and peroxisome, while IDH2 and IDH3 are located in mitochondria^3^. *IDH1* mutations were first implicated in carcinogenesis by a high-throughput study of the mutational landscape of breast and colorectal cancers^4^. Since then, mutations in *IDH1* or *IDH2* genes have been detected in many different tumors, primarily gliomas (>80% of grade II and grade III gliomas)^5^, acute myeloid leukemia (AML, ~15% of cases)^6,7^ and chondrosarcomas (~50% of cases)^8^. *IDH* mutations have been reported, albeit with a lower prevalence, in thyroid carcinoma (5–15% of cases)^9,10^, cholangiocarcinoma (15–20% of cases)^11^, and other solid neoplasms^12–15^. Among brain tumors *IDH* mutations are identified in over 80% of grade II and grade III gliomas (astrocytomas, oligodendrogliomas)^16,17^ and in about 5% of glioblastomas (GBM)^16^. According to the 2016 World Health Organization (WHO) classification of Central Nervous System tumors, establishing whether a brain tumor is *IDH* mutated or wild-type (WT) is a crucial requisite for the classification of gliomas^18^.

The large majority of *IDH1* cancer-associated mutations affect codon 132, resulting in the amino acidic arginine(R)-to-histidine(H) substitution (p.R132H, c.395 G > A). Mutations other than p.R132H are found with a lower frequency, such as p.R132C (c.394 C > T), p.R132S (c.394 C > A), p.R132G (c.394 C > G) or p.R132L (c.395 G > T)^5,17,19–21^. However, other mutations not involving codon 132 have also been detected^5^. IDH-R132 mutations, as well as other *IDH1* and *IDH2* mutations (such as IDH1-G97D, IDH1-Y139D, IDH2-R172, IDH2-R140) have been shown to produce the 2-hydroxyglutarate (2HG) oncometabolite, while other rare mutations (e.g. IDH1-H133Q, IDH1-I130M, IDH1-G123R, IDH1-I99M, IDH1-V178I, IDH1-V71I) result in decreased IDH activity without a concomitant increase in 2HG production^22^.

Usually, synonymous single nucleotide polymorphisms (SNPs) do not change protein function, insofar as the amino acid sequence of the protein is not affected by the nucleotide change. Some silent SNPs, however, may lead to a protein defect, for example when they are localized in a splicing site^23,24^. In the case of the *IDH1* gene, Wagner *et al*. (2010) found a silent SNP in a cohort of cytogenetically normal AML samples, that changes codon 105 of exon 4 from “GGC” (Gly) to “GGT” (Gly)^25^. The SNP (p.G105G, rs11554137:C > T -IDH1^105GGT^, minor allele frequency 0.0569) has since been frequently reported in AML and is linked to an adverse prognosis^26–28^. It has also been reported in brain tumors in a study of patients with gliomas (grade II to IV) from France and Germany^29^ and in a series of Bulgarian GBM patients^30^, as well as in thyroid tumors (both carcinomas and adenomas)^9,10^.

The role and biologic significance of the *IDH1*^105GGT^ SNP in tumorigenesis is poorly understood, but it appears to be associated with increased *IDH1* mRNA levels leading to altered NADPH production^25,29^.

The aim of the present study was to assess the prevalence of the *IDH1*^105GGT^ SNP in a cohort of Italian patients with brain tumors classified according to 2016 WHO criteria, and investigate its association with clinicopathologic features and *IDH* tumor missense mutations.

## Results

**Figure 1:**
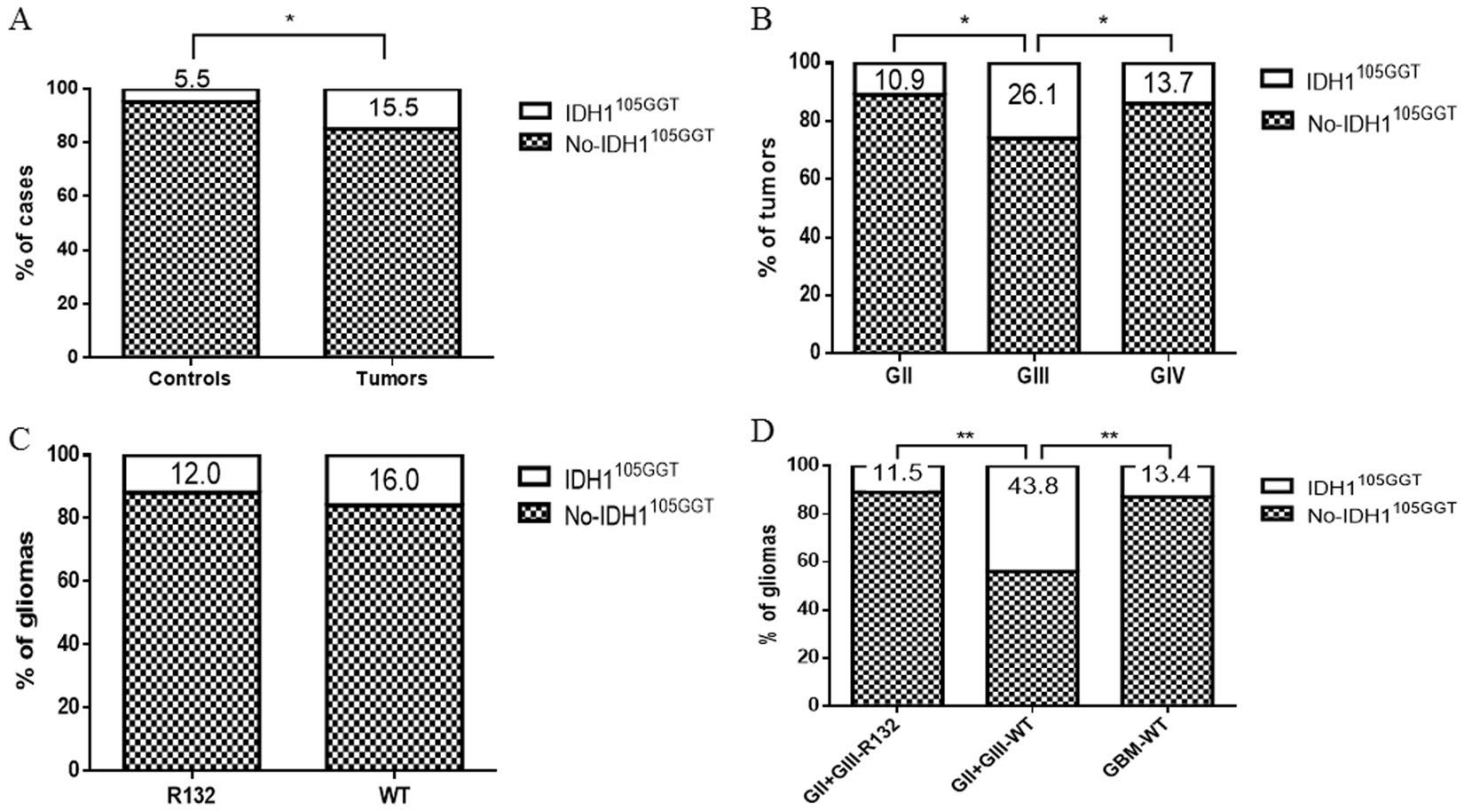
Prevalence of *IDH*^*105GGT*^. (**A**) Comparison of *IDH*^*105GGT*^ between controls and patients with brain tumors. (**B**) *IDH*^*105GGT*^ in grade II, III and IV brain tumors. (**C**) *IDH*^*105GGT*^in gliomas with and without *IDH* tumor missense mutation. (**D**) *IDH*^*105GGT*^ in grade II and III gliomas with and without *IDH* tumor missense mutation and in grade IV without *IDH* tumor missense mutation. *IDH*^*105GGT*^: cases with *IDH*^*105GGT*^; No-*IDH*^*105GGT*^: cases without the *IDH*^*105GGT*^*;* GII: grade II brain tumors; GIII: grade III brain tumors; GIV: grade IV brain tumors; GBM: glioblastoma; R132: cases with IDH-missense mutation; WT: wild type.

NGS primers allowed a reliable analysis of the nucleotide sequence of codon 105 in all samples. Overall, *IDH1*^105GGT^ was found in 44 of 293 (15.0%) enrolled tumors (Table 1) and in 6 of 109 (5.5%) control individuals (*p* = *0*.*0102*) (Fig. 1A).

**Table 1:** Histological classification of the tumor samples analyzed and distribution ofIDH1^105GGT^. All the oligodendrogliomas harboured a mutation in *IDH1* or *IDH2* genes and showed co-deletion of chromosome arms 1p/19q. WT: Wild Type; °IDH1^105GGT^was in the homozygous state.

Diagnosis	N° of cases	IDH1^105GGT^ (%)
**Grade II tumors**	**64**	**7 (10**.**9)**
***Astrocytomas***	**34**	**4 (11**.**8)**
Diffuse astrocytoma, IDH-WT	6	2 (33.3)
Diffuse astrocytoma, IDH-mutant	25	1 (4)
Gemistocytic Astrocytoma, IDH-mutant	1	1 (100)
Pleomorphicxanthoastrocytoma, IDH-WT	2	0 **(**−)
***Oligodendrogliomas***	**25**	**2 (8)**
Oligodendroglioma, IDH-mutant and 1p/19q codeleted	25	2° (8)
***Other Grade II brain tumors***	**5**	**1 (20)**
Ependymoma	4	0 (−)
Neurocytoma	1	1 (100)
**Grade III tumors**	**46**	**12** (**26**.**1)**
***Anaplastic Astrocytomas***	***29***	***9*** (***31***.***0)***
Anaplastic astrocytoma, IDH-WT	7	4 (57.1)
Anaplasticastrocytoma, IDH-mutant	21	4 (19)
Anaplastic Pleomorphic xanthoastrocytoma, IDH1-WT	1	1 (100)
***AnaplasticOligodendrogliomas***	***15***	***2*** (***13***.***3)***
Anaplastic oligodendroglioma, IDH-mutant and 1p/19q codeleted	15	2 (13.3)
***Other Grade III brain tumors***	***3***	***1*** (***33***.***3)***
Anaplastic ependimoma	2	1 (50.0)
**Grade IV**	**183**	**25** (**13**.**7)**
Glioblastoma, IDH-WT	179	24° (13.4)
Glioblastoma, IDH-mutant	4	1 (25.0)

In all but two patients harboring *IDH1*^105GGT^, the SNP was detected in about 50% of alleles analyzed (range: 45–53%), a frequency that is fully compatible with a heterozygous germline event. In the other two patients, *IDH1*^105GGT^ was detected in 100% of the alleles analyzed, compatible with a homozygous germline condition.

### *IDH1*^105GGT^and histological grade

Seven of 64 (10.9%) grade II cases (including 59 gliomas, 4 ependymomas and 1 neurocytoma) harbored *IDH1*^*105GGT*^ (Fig. 1B), all but one in the heterozygous state. In one case (oligodendroglioma, IDH1-mutated and 1p/19q co-deleted), the SNP was detected in 100% of the alleles analyzed, compatible with a homozygous condition (Table 1). Among grade II gliomas, 6 of 59 (10.1%) harbored *IDH1*^*105GGT*^.

Twelve of 46 (26.1%) grade III tumors (including 43 gliomas and 3 anaplastic ependymomas) harbored *IDH1*^*105GGT*^, all in the heterozygous condition (Fig. 1B). Among grade III gliomas, 11 of 43 (26.6%) harbored *IDH1*^*105GGT*^ (Table 1).

As regards grade IV tumors, 25 of 183 (13.7%) GBM were positive for IDH1^105GGT^ (Fig. 1B), all but one (a GBM-IDH WT) in the heterozygous state.

We found a statistically significant difference in the prevalence of *IDH1*^105GGT^ between tumor grades, with the highest frequency among tumors belonging to grade III, compared to grades II and IV (*p* = *0*.*038* and *p* = *0*.*041* respectively, Chi-squared test, Fig. 1B). Also among gliomas, *IDH1*^105GGT^ is more frequent in grade III than in grade II or IV cases (*p* = *0*.*039* and *p* = *0*.*046*, respectively). No statistically significant difference in prevalence was observed between grades II and IV, for both tumors and gliomas (p = 0.5765 and p = 0.6546, respectively, Chi-squared test).

### *IDH1*^105GGT^ and other *IDH* mutations

We observed an *IDH1* or *IDH2* mutation in 51 of 64 (79.7%) grade II tumors (all 51 cases were gliomas and 37 of these harbored the common p.R132H *IDH1* mutation), in 36 of 46 (78.3%) grade III tumors (all 36 cases were gliomas and 31 harbored p.R132H), and in 4 of 183 (2.2%) grade IV tumors (all p.R132H).

Among 91 *IDH*-mutated gliomas, 11 (12.1%) also carried *IDH1*^*105GGT*^. In 31 gliomas, *IDH1*^*105GGT*^ was detected in the absence of any *IDH* missense mutation. In accordance with data previously reported^29^, we found no correlation between the presence of *IDH* missense mutations in the tumor and the presence of *IDH1*^*105GGT*^ (p = 0.4749, Fisher’s exact test) (Fig. 1C). However, *IDH1*^*105GGT*^ was more frequent in grade II and III gliomas without than with *IDH* missense tumor mutations (43.8% vs 11.5% respectively – *p* = *0*.*005*, Fisher’s exact test) (Fig. 1D). The SNP was also more frequent in grade II and III gliomas lacking *IDH* missense mutations than in GBM lacking *IDH* missense mutations (43.8% vs. 13.4%, *p* = *0*.*005*, Fisher’s exact test) (Fig. 1D).

### *IDH1*^105GGT^ and histological subtypes

In grade II and III tumors, *IDH1*^105GGT^ was more frequent in astrocytomas (13 of 63 cases −20.6%) than in oligodendrogliomas (4 of 40 cases −10%), but the difference did not reach statistical significance (p = 0.1837, Fisher’s exact test). Even after the inclusion of GBMs in the astrocytoma group (38 of 246–15.4%), the prevalence of *IDH1*^105GGT^ was not statistically different between the oligodendroglial and astrocytic lineages (p = 0.4743, Fisher’s exact test).

### *IDH1*^105GGT^ and age

**Figure 2:**
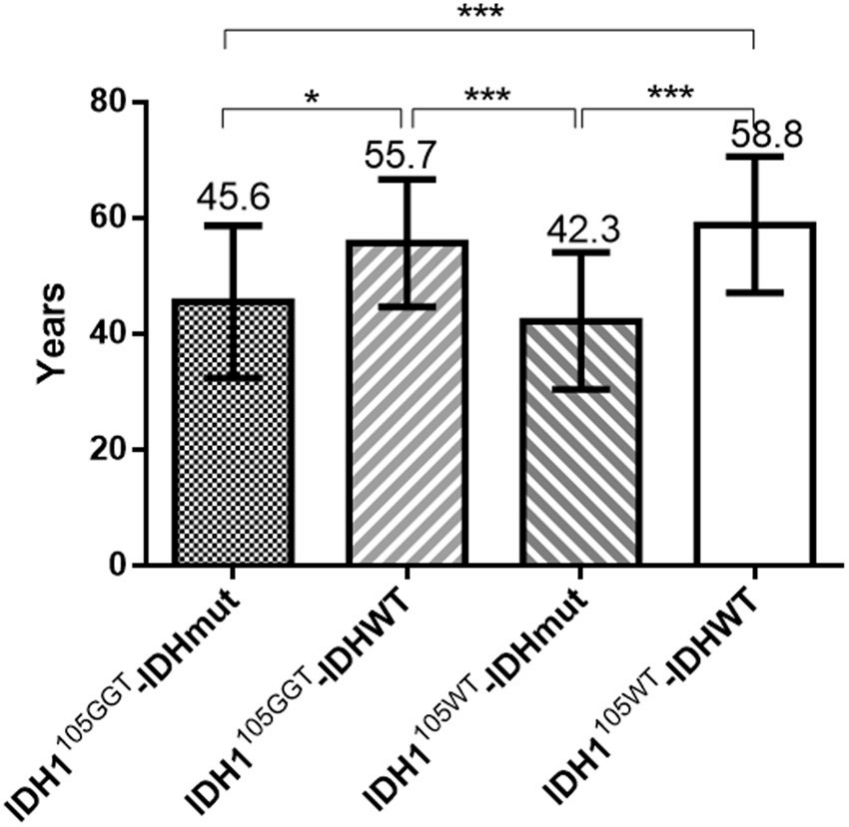
Age distribution of patients according to the presence of *IDH1*^*105GGT*^ and *IDH* missense tumor mutations. IDH1^105GGT^: patients with tumor with IDH1^105GGT^; IDH1^105WT^: patients with tumor without IDH1^105GGT^; IDHmut: patients with tumor with *IDH* missense mutation; IDHWT: patients with tumor without *IDH* missense mutation. *p < 0.05 (Tukey’s multiple comparisons test); ***p < 0.001 (Tukey’s multiple comparisons test). Bars represent standard deviation (SD).

The age of patients harboring *IDH1*^*105GGT*^ ranged from 26 to 74 years (mean 51.7ys). These patients were slightly younger than those without the SNP (mean age 53.2ys; age range: 17–84ys), but the difference was not statistically significant (p = 0.4476, Mann Whitney test). Figure 2 summarizes the statistical relationship between age, *IDH1*^*105GGT*^ and *IDH* missense tumor mutations. There are significant differences among patient age and the distribution of *IDH1*^*105GGT*^ and that of *IDH* missense tumor mutations. In particular, patients bearing only the SNP (mean age 55.7 years) were older that patients bearing both the SNP and *IDH* missense tumor mutations (mean 45.6ys, p < 0.05, Tukey’s multiple comparisons test), or those bearing only missense mutations (mean 42.3ys, p < 0.001, Tukey’s multiple comparisons test).

## Discussion

In our Italian cohort, the prevalence of the *IDH1*^*105GGT*^ SNP was considerably higher in patients with brain tumors compared to the control population (15.5% vs 5.5%, respectively).

Few studies have analyzed *IDH1*^*105GGT*^ in brain tumors, likely because routine molecular pathology methods do not always allow its identification. *IDH1*^*105GGT*^ status cannot be inferred by immunohistochemical methods, or by the mutation-specific PCR assays commonly used to diagnose p.R132H. Furthermore, sequencing requires the design of specific primers to include codon 105. Our NGS primers allowed us to reliably diagnose the SNP genotype in all samples.

Wang *et al*. in a cohort of French and German patients with gliomas, did not find a statistical correlation between *IDH1*^105GGT^ and tumor histological grade^29^. In our cohort of Italian patients, we found a statistical association of *IDH1*^*105GGT*^ with grade III gliomas, in particular with grade III astrocytomas. Importantly, among grade II and III gliomas, *IDH1*^*105GGT*^ was more frequent in those cases without *IDH* missense tumor mutations (Fig. 1D). Previous studies reported this polymorphism as an adverse prognostic factor in patients with acute myeloid leukemia^25^; findings in the series of Wang *et al*. suggested a strong association with adverse outcome in patients with malignant glioma^29^. No association of the *IDH1*^105GGT^ SNP with survival was found in the GBM series of Stancheva *et al*.^30^.

Although the functional effects of this polymorphism are still unclear, prediction analysis has shown that nucleotide 315 of the *IDH1* gene may be within a putative Exonic Splicing Silencer (ESS) motif (ESRsearch Tool, http://esrsearch.tau.ac.il/)^31^. A nucleotide substitution in this region could lead to a protein defect due to incorrect regulation of constitutive or alternative splicing. Moreover, *IDH1*^*105GGT*^ may be in linkage disequilibrium with other “tumor predisposing” variants.

Current opinion favors the existence of two major glioma groups: *IDH*-mutant gliomas, that are typically grade II and III tumors with a relatively favorable prognosis and *IDH-*WT tumors with a worse prognosis. As the large majority of *IDH-*WT tumors are grade IV, some authors have suggested that *IDH*-WT astrocytomas are in fact under-sampled *IDH-*WT GBMs and that they should be treated accordingly^32^. However, some subsets of *IDH*-WT low-grade gliomas do not have the molecular characteristics of GBM. These tumors likely represent other entities on a biological level. Some *IDH-*WT astrocytomas correspond to so called “pediatric type” tumors, sharing genetic and epigenetic features with pilocytic astrocytomas^33^.

In this context, the *IDH1*^105GGT^ SNP may represent an important marker to further dissect and understand the clinical and biological features of *IDH*-WT infiltrating gliomas. Additional studies are warranted to clearly define the genetic profile and clinical outcome of patients with the *IDH1*^105GGT^ SNP.

## Methods

### Case selection

A total of 293 consecutive cases of primitive brain tumors (64 grade II tumors, 46 grade III tumors, 183 grade IV tumors) were retrieved from the archives of Anatomic Pathology of Bellaria Hospital (Bologna, Italy). Samples were diagnosed and reclassified according to 2016 WHO criteria^18^. Patients were 181 males (61.8%) and 112 females (38.2%), aged from 17 to 84 years (mean age 52.9ys). Control DNA samples were analyzed from the peripheral blood of 109 individuals who underwent blood testing at the same institution to infer the prevalence of *IDH1*^105GGT^ in the reference population. None of the controls was affected by brain tumor or other neoplastic diseases. The study was approved by Ethic Committee of Azienda Sanitaria Locale di Bologna (protocol number CE09113 of 29^th^ September 2013, Bologna, Italy). All information regarding the human material was managed using anonymous numerical codes and all samples were handled in compliance with the Helsinki Declaration (https://www.wma.net/policies-post/wma-declaration-of-helsinki-ethical-principles-for-medical-research-involving-human-subjects/).

### IDH1 and IDH2 analysis

All analyses were performed on DNA from formalin fixed and paraffin embedded (FFPE) specimens, extracted with the QuickExtract FFPE DNA Extraction Kit (Epicentre, Madison, WI, U.S.A.). Control DNA from blood specimens were extracted using the MasterPure DNA Purification Kit (Epincentre, Madison WI, USA). *IDH1* (exon 4, codons 96–138) and *IDH2* (exon 4, codons 151–178) amplicons were generated using the following primers: IDH1 Fw 5′-GAAACAAATGTGGAAATCACCA-3′, IDH1 Rv 5′-TCACATTATTGCCAACATGACT-3′; IDH2 Fw 5′-AGCCCATCATCTGCAAAAA-3′, IDH2 Rv 5′-TGTGGCCTTGTACTGCAGA-3′. The *IDH1*^105GGT^ SNP (rs11554137) is 27 codons (81 nucleotides) upstream of the *IDH1* hot spot codon (p.R132), well within the DNA region amplified by our set of primers.

Sequencing was performed using the 454 GS-Junior next generation sequencer (NGS) (Roche Diagnostic, Mannheim, Germany) according to established protocols (http://www.454.com/)^34^.

Categorical variables were compared using the Chi-square test or Fisher’s exact test. Continuous variables were compared using the Mann-Whitney test. Statistical comparison among *IDH1* alterations and age was determined by the one-way analysis of variance (ANOVA) with Tukey’s multiple comparison test. A p-value < 0.05 was considered as statistically significant. Statistical analyses were performed using GraphPad Prism 6.01 (GraphPad Software).

### Data availability

The datasets analysed during the current study are available from the corresponding author on reasonable request.
